# First Report and Integrated Characterization of *Aeromonas veronii* Associated with the Protected Fish *Diptychus maculatus* in Xinjiang, China

**DOI:** 10.3390/ani16172787

**Published:** 2026-09-04

**Authors:** Syeda Maira Hamid, Huale Lu, Huimin Hao, Kaipeng Zhang, Wentao Zhu, Jie Wei, Zhulan Nie

**Affiliations:** 1 College of Life Science and Technology, Tarim University, Alar 843300, China; smairahamid@gmail.com (S.M.H.); 18955281085@163.com (H.L.); 10757223077@stumail.taru.edu.cn (H.H.); zkp19980604@163.com (K.Z.); zhuwt@taru.edu.cn (W.Z.); 120070006@taru.edu.cn (J.W.); 2 Xinjiang Production and Construction Corps Key Laboratory of Protection and Utilization of Biological Resources in Tarim Basin, Alar 843300, China

**Keywords:** *Diptychus maculatus*, *Aeromonas veronii*, antimicrobial resistance, biofilm formation, aquatic bacterial pathogen, molecular identification, wildlife disease surveillance, conservation breeding, freshwater microbiology

## Abstract

*Aeromonas veronii* is a widely distributed opportunistic aquatic pathogen associated with diseases in freshwater fish. Despite the ecological and conservation significance of *Diptychus maculatus*, a protected cold-water fish inhabiting high-altitude ecosystems, information regarding its associated bacterial communities remains limited. This study aimed to isolate and characterize *A. veronii* recovered from *D. maculatus* and provide baseline information on its occurrence and phenotypic characteristics. Eight bacterial isolates were recovered from various tissues, including skin, gills, eye, intestine, dorsal fin, body kidney, gonad, and spleen of randomly sampled fish individuals from Xinjiang, China. Phenotypic and biochemical characterization, together with 16S rRNA sequencing, supported their identification as *A. veronii*, while *gyrB* analysis of a representative isolate provided additional species-level confirmation. Antimicrobial susceptibility testing revealed a consistent multidrug-resistance phenotype among all isolates. The isolates were susceptible to enrofloxacin, cefotaxime, ceftriaxone, and florfenicol. Intermediate responses were observed for ciprofloxacin, ofloxacin, doxycycline, oxytetracycline, and trimethoprim–sulfamethoxazole, whereas resistance was detected against norfloxacin, neomycin, penicillin, amoxicillin, tetracycline, and erythromycin. In vitro biofilm assays demonstrated weak to moderate biofilm-forming capacity among isolates. These findings provide baseline data for wildlife microbial surveillance and conservation-oriented monitoring of protected fish populations, supporting future investigations into environmental monitoring, genomic characterization, and host–microbe interactions.

## Simple Summary

*Diptychus maculatus* is a second-class protected cold-water fish inhabiting ecologically sensitive high-altitude aquatic ecosystems. Yet, there is limited information on the bacteria associated with this species. In this study, bacteria were recovered from multiple tissues of *D. maculatus* and characterized based on phenotypic, biochemical, and molecular analyses. The recovered isolates were identified as *Aeromonas veronii* based on phenotypic characterization and 16S rRNA gene sequencing, with additional *gyrB* confirmation performed for a representative isolate. As *A. veronii* is an important opportunistic aquatic pathogen, its antimicrobial susceptibility profile and biofilm-forming ability were evaluated. The isolates exhibited similar responses to the tested antimicrobial agents and demonstrated weak-to-moderate biofilm formation under laboratory conditions. These findings offer baseline data on the occurrence and phenotypic traits of *A. veronii* in a protected fish species and contribute to conservation-oriented wildlife microbial surveillance in high-altitude aquatic ecosystems. The study highlights the need for future investigations into host-associated microbial diversity, microbial ecology, genomics and conservation-oriented monitoring of protected fish populations to gain a better understanding of their ecological and health-related implications.

## 1. Introduction

Freshwater fish are significant components of aquatic biodiversity and ecosystem functioning, providing ecosystem stability, nutrient cycling, and aquatic ecosystem services. However, habitat degradation, environmental pollution, alteration of hydrological conditions, climate change, and emerging infectious diseases are among the various threats that are causing population declines in freshwater fish throughout the world [1]. Maintaining host health has become a crucial component of modern conservation biology, alongside habitat protection and population restoration. Microorganisms associated with aquatic organisms can influence host physiology, immune response, environmental interactions and susceptibility to opportunistic infections [2]. Therefore, microbial surveillance has gained increasing attention as a valuable approach to investigate and understand host-associated bacterial communities and support conservation efforts for threatened and protected fish species.

Among vulnerable freshwater fishes, *Diptychus maculatus*, a cold-water Schizothoracine fish, represents an ecologically important species that is widely distributed in high-altitude inland river systems of Central Asia and Northwest China [3]. Phylogeographic analysis has identified this species as being closely linked to river systems in the North of the Qinghai–Tibetan Plateau, reflecting its adaptation to cold freshwater environments [4]. Due to the population decline associated with habitat alteration, anthropogenic disturbance, habitat fragmentation, and disruption of natural migration routes, *D. maculatus* has been classified as a nationally protected second-class protected species in China [3].

Most conservation efforts for this species have primarily focused on population growth, artificial propagation, and habitat restoration. However, understanding the microbial characteristics associated with protected fish populations is also important because host-associated microbes may influence fish health, environmental adaptation, and long-term conservation outcomes [5]. Despite increasing interest in fish-associated microbiota, microbiological information regarding *D. maculatus* remains limited. Previous research has mainly focused on intestinal microbial communities, evolutionary relationships and population biology [6,7,8], while the bacteria associated with anatomical tissues remain comparatively poorly characterized. This information gap limits understanding of the bacterial diversity related to this protected species and underlines the importance of systematic characterization of potentially important microbial taxa associated with conservation populations [9,10,11].

The genus *Aeromonas* comprises Gram-negative, facultative anaerobic bacteria found in many freshwater ecosystems. Members of this genus inhabit a wide range of ecological niches and include species capable of acting as opportunistic pathogens of aquatic animals and humans [12,13]. Motile *Aeromonas* have been reported to cause septicemia, ulcerative lesions, tissue infection and systemic disease in cultured and wild fish under favorable environmental and host conditions [14]. Among these species, *Aeromonas veronii* has received considerable attention due to its widespread distribution in nature, adaptability to a wide range of hosts, genetic diversity, and behavioral traits that support its persistence in aquatic habitats [15,16]. *A. veronii* has been reported in many freshwater fish species, such as crucian carp (*Carassius auratus*) [17], common carp (*Cyprinus carpio*) [18], channel catfish (*Ictalurus punctatus*) [19], grass carp (*Ctenopharyngodon idella*) [20] and rohu (*Labeo rohita*) [21]. The findings demonstrate that *A. veronii* is an ecologically relevant freshwater-associated bacterial species and highlight the significance of studying the occurrence of this species in less-studied protected fish populations [22].

Accurate identification of *Aeromonas* species remains challenging because closely related species frequently exhibit similar and overlapping phenotypic traits. Conventional biochemical approaches alone may not always provide sufficient resolution for reliable species-level identification within this genus. To provide better taxonomic resolution of *Aeromonas* isolates, molecular techniques, in particular 16S rRNA gene sequencing and additional housekeeping gene analysis, such as *gyrB,* have, therefore, been increasingly applied. Phenotypic characterization in conjunction with molecular identification offers a more reliable approach for investigating bacterial occurrences in aquatic hosts.

In aquatic bacteria, antimicrobial susceptibility and biofilm formation are two phenotypic traits that are frequently evaluated. Aquatic environments can potentially contribute to the persistence and dissemination of antimicrobial-resistant bacteria, and the resistance patterns of *A. veronii* isolates can differ based on environmental factors, host species, and anthropogenic factors [7,12,14]. Characterization of antimicrobial susceptibility profiles provides baseline information regarding resistance phenotypes and may contribute to understanding the bacterial characteristics associated with aquatic organisms. Moreover, biofilm formation represents an adaptive bacterial ability that contributes to attachment, survival and persistence in response to changing environmental conditions [16]. Therefore, complementary phenotypic information regarding recovered isolates can be obtained from antibiotic susceptibility profiles and biofilm-forming capacity.

Although *A. veronii* has been reported from various freshwater organisms, information regarding its association with protected high-altitude cold-water fishes, particularly *D. maculatus*, remains limited. Therefore, the present study was designed to isolate and characterize *A. veronii* associated with *D. maculatus* by employing conventional bacteriological isolation and characterization methods, 16S rRNA gene sequencing and confirmatory *gyrB* sequencing of a representative isolate. Furthermore, antimicrobial susceptibility profiles and biofilm formation capacity were evaluated to provide baseline phenotypic information regarding recovered bacterial isolates. This study focuses on bacterial characterization and baseline health-related microbial surveillance, rather than establishing disease causation, providing essential information for future research into the host-associated ecology, microbial interactions, genomic characterization and conservation management of fish health.

## 2. Materials and Methods

### 2.1. Fish Sampling and Ethical Considerations

**Figure 1:**
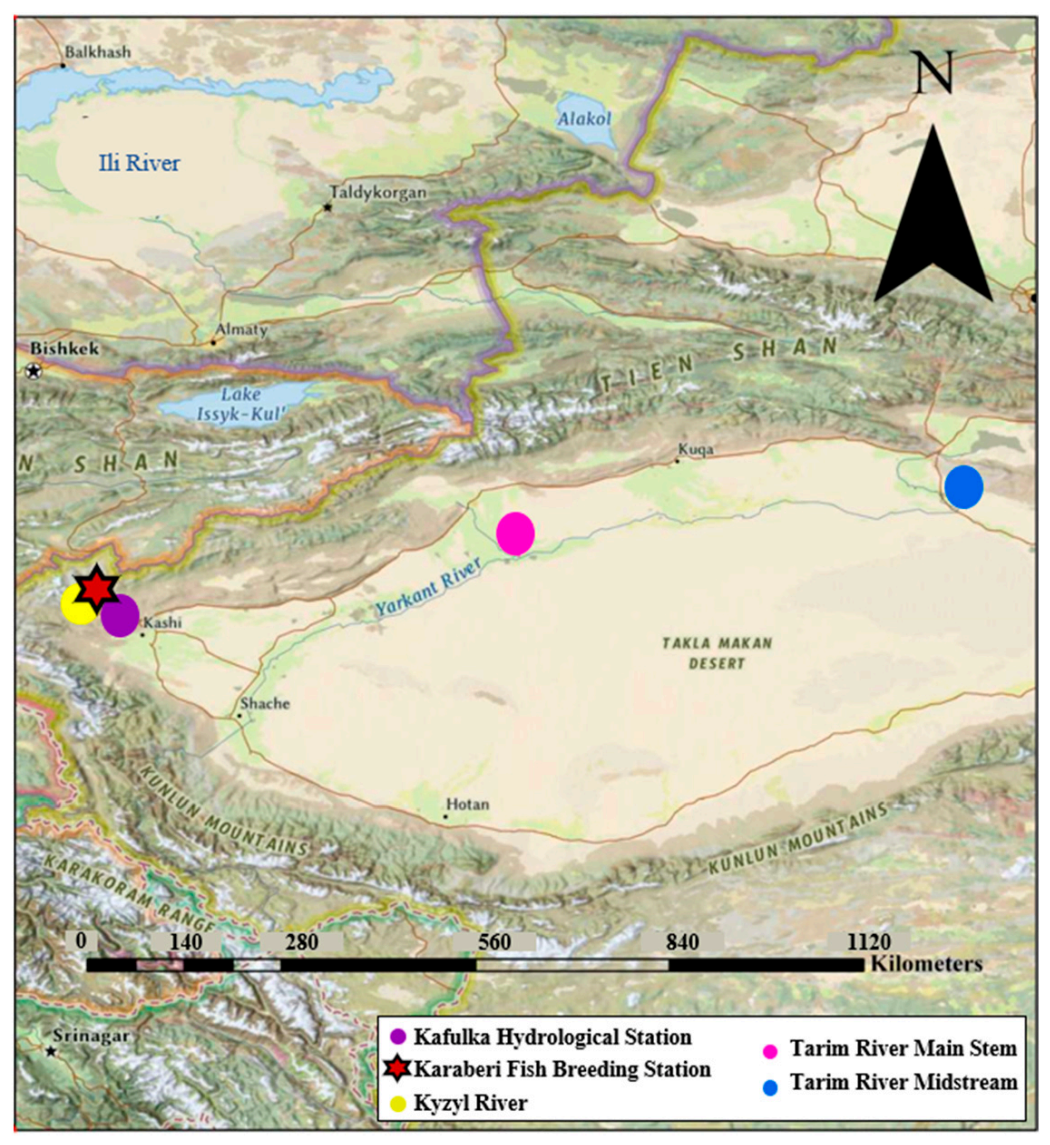
Geographical location of the Karaberi Fish Breeding and Release Station, the sole sampling location in the present study, situated on the Karaberi River, a major tributary of the Kyzyl River within the Tarim River system, Xinjiang, China.

A total of 55 specimens of *D. maculatus* were collected from the Karaberi Fish Breeding and Release Station located on the Karaberi River, a main tributary of the Kyzyl River within the Tarim River system, in Xinjiang Uygur Autonomous Region, China. The sampling site was located at around 39.35° N latitude and 75.15° E longitude, with an altitude of 1775–1777 m above sea level (Figure 1). The facility had experienced mortality events involving *D. maculatus* individuals. Sampling was conducted in June 2024 during a field investigation, and fish exhibiting apparent external abnormalities during observation were selected for bacteriological examination. During external examination, some fish exhibited localized reddish discoloration around the head and ventral body surface, together with variation in overall body coloration [23]. Fish were collected alive and transported to the laboratory in oxygen-filled plastic bags under temperature-controlled conditions for about 7–8 h (600–640 km).

During temporary holding, fish were maintained at approximately 10–15 °C at a stocking density of 1 individual per 5–10 L of water, under constant aeration with no feeding provided during the short-term observation [5]. The pH of the water held in the system was maintained between 7.7 and 8.5. An identification number was assigned to each specimen, and biometric parameters such as body length and body weight were measured (Supplementary Table S1). Fish were euthanized using tricaine methanesulfonate (MS-222; Argent Chemical Laboratories, Inc., Redmond, WA, USA) at 100 mg/L before dissection [24]. The animal study protocol was approved by the Institutional Review Board of Tarim University (Approval No. PB20241227003), and all procedures were conducted in accordance with institutional and national guidelines for the care and use of animals.

### 2.2. Sample Collection and Bacterial Isolation

Following external examination, 10 fish selected for bacteriological examination were dissected under aseptic conditions in a laminar flow cabinet. Fish surfaces were disinfected with 70% ethanol prior to dissection, and tissue samples were collected using sterile instruments. Equipment was sterilized between successive tissue samples to minimize potential cross-contamination. Individual samples were collected from the eye, intestine, dorsal fin, body kidney, anus, skin, liver, gill, gonad, and spleen. Each tissue sample was linked to the corresponding fish identification number, and recovered isolates were assigned unique codes (Supplementary Table S1).

Collected tissue samples were homogenized in sterile phosphate-buffered saline (PBS; 1×, pH 7.4; Biosharp, Hefei, China). Serial dilutions (10^−1^–10^−6^) were prepared to facilitate recovery of well-separated colonies for bacterial isolation. Aliquots from selected dilutions were inoculated onto tryptic soy agar (TSA; Qingdao Hope Biotechnology Co., Ltd., Qingdao, China) in triplicate and incubated aerobically at 32 °C for 24–48 h to recover culturable mesophilic aquatic bacteria, including *Aeromonas* spp. [19,21,25,26]. Representative colonies were selected from culture-positive samples based on colony morphology and subsequently purified by repeated streaking on fresh TSA plates until pure cultures were obtained. Uninoculated TSA plates containing sterile PBS were used as sterility controls during bacterial isolation. Eight purified isolates (DMNZ01–DMNZ08), each linked to a specific fish individual and tissue source, were retained for subsequent characterization. Pure cultures were maintained on TSA slants at 4 °C for short-term storage and preserved in tryptic soy broth (TSB) supplemented with 20% glycerol at −80 °C for long-term storage [17].

### 2.3. Phenotypic and Biochemical Characterization

All eight purified bacterial isolates (DMNZ01–DMNZ08) were initially characterized based on colony morphology, such as size, shape, color, margin and elevation on TSA plates after incubation. The cellular morphology and Gram reactions of the bacteria were determined using the standard Gram-staining procedure with a commercial staining kit (BKMAM Biotechnology Co., Ltd., Changsha, China), and the stained smears were examined under a light microscope (A53; Motic China Group Co., Ltd., Hong Kong, China) at a magnification of 1000× with immersion oil.

Biochemical characterization was performed in triplicate using a ready-to-use commercial bacterial identification system (Guangdong Huankai Biology Sci & Tech Co., Ltd., Guangzhou, China) following the manufacturer’s instructions [26]. The biochemical tests performed included indole production, methyl red (MR), Voges–Proskauer (VP) reaction, citrate utilization, enzymatic and metabolic activities, such as MUG, lysine decarboxylase, and ornithine decarboxylase, and carbohydrate utilization (lactose, raffinose, cellobiose, and sorbitol). Motility was evaluated using the motility medium provided in the identification system according to the manufacturer’s instructions. Results were interpreted based on the color changes and growth patterns described in the kit guidelines. The reference strain *A. veronii* BNCC138468 was included as a comparative strain for biochemical profile evaluation. All purified isolates were phenotypically and biochemically characterized, while the species-level identification was further confirmed by molecular approaches, including 16S rRNA gene sequencing and *gyrB* analysis.

### 2.4. Molecular Identification by 16S rRNA Gene Sequencing and gyrB Amplification

Genomic DNA was extracted from fresh overnight bacterial cultures using a UNIQ-10 column DNA extraction kit (Sangon Biotech (Shanghai) Co., Ltd., Shanghai, China), as per the manufacturer’s protocol. Universal bacterial primers 27F (5′-AGAGTTTGATCCTGGCTCAG-3′) and 1492R (5′-GGCTACCTTGTTACGACTT-3′) [26] were used to amplify the 16S rRNA gene. For additional species-level confirmation, the *gyrB* gene of the representative isolate (DMNZ06) was amplified using the primer *gyrB* (5′-GAAGTCATCATGACCGTTCTGCA(TC)GC(TCAG)GG(TCAG)GG(TCAG)AA(AG)TT(TC)GA-3′) and *gyrB*-R (5′-AGCAGGGTACGGATGTGCGAGCC(AG)TC(TCAG)AC(AG)TC(TCAG)GC(AG)TC(TCAG)GTCAT-3′) according to previously described protocols [7,26,27,28,29]. PCR amplification of both the 16S rRNA and *gyrB* targets was performed in a 50 µL reaction mixture using TransStart FastPfu DNA Polymerase in a Thermal Cycler (Applied Biosystems, Foster City, CA, USA). Following the optimization, the same thermal-cycling program was applied to both targets, consisting of an initial denaturation at 95 °C for 5 min, 30 cycles of denaturation at 95 °C for 30 s, annealing at 55 °C for 30 s, and extension at 72 °C for 45 s, and a final extension at 72 °C for 10 min, consistent with previously reported amplification procedures for 16S rRNA and *gyrB* analysis [29,30]. The amplified products were verified by agarose gel electrophoresis (Tsingke Biotechnology, Beijing, China). The 16S rRNA amplicons and the *gyrB* amplicon of representative isolate DMNZ06 were subsequently sequenced using an ABI 3730XL DNA Analyzer (Applied Biosystems) [16,20,26]. The obtained sequences were trimmed and assembled, and taxonomic identification was performed by comparing the obtained sequences with the reference sequences in the NCBI GenBank database using BLASTn (https://blast.ncbi.nlm.nih.gov/Blast.cgi?PROGRAM=blastn&PAGE_TYPE=BlastSearch&LINK_LOC=blasthome, accessed on 25 April 2026). Phylogenetic analysis was performed using the neighbor-joining method implemented in MEGA X software (version 12.1) with 1000 bootstrap replications. Bootstrap values > 70% were considered statistically supported [26].

### 2.5. Antimicrobial Susceptibility Testing

Antimicrobial susceptibility of all bacterial isolates was determined using the Kirby–Bauer disk diffusion method on Mueller–Hinton agar (MHA; BaseBio, Hangzhou, China) following standardized inoculum preparation, disk diffusion procedures, and inhibition-zone measurement described in Clinical and Laboratory Standards Institute (CLSI) guidelines, with reference to previously described methods for aquatic bacterial isolates [21,27,31,32,33,34]. A total of 15 different antimicrobial discs (Hangzhou Microbial Reagent Co., Ltd., Hangzhou, China) representing different antimicrobial classes were tested, including enrofloxacin (5 µg), ciprofloxacin (5 µg), norfloxacin (10 µg), ofloxacin (5 µg), neomycin (30 µg), penicillin (10 IU), amoxicillin (10 µg), cefotaxime (30 µg), ceftriaxone (30 µg), tetracycline (30 µg), doxycycline (30 µg), oxytetracycline (30 µg), trimethoprim–sulfamethoxazole (1.25/23.75 µg), erythromycin (15 µg), and florfenicol (30 µg).

Fresh bacterial cultures were adjusted to a 0.5 McFarland (approx. 1 × 10^8^ CFU/mL), and 100 µL of standardized bacterial suspension was uniformly spread on the surface of the MHA plates with the help of sterile cotton swabs. Each antimicrobial agent was tested separately, with a single antimicrobial disc applied to each inoculated Mueller–Hinton agar (MHA) plate [30]. The plates were incubated aerobically at 32 °C for 24 h, a condition that provided uniform lawn formation, stable bacterial growth, and clearly measurable inhibition zones, consistent with previously described procedures for mesophilic aquatic bacteria, including *Aeromonas* spp. [21,27,31,32,33]. *A. veronii* BNCC138468 (BeNa Culture Collection, Beijing, China) was included as a reference strain and tested under identical experimental conditions to allow for comparative evaluation of susceptibility profiles between the recovered isolates and the reference strain.

After incubation, inhibition zone diameters were measured using a vernier caliper (Guanglu, Guilin, China), and isolates were categorized as susceptible, intermediate or resistant. Since no single interpretive guideline was available to cover the entire antimicrobial panel tested for aquatic *A. veronii* in this study, susceptibility categories were assigned based on previously reported criteria for *Aeromonas* spp. and *A. veronii*, considering the antimicrobial agent, disc concentration, and corresponding interpretive references [21,23,24,31,35,36].

The inhibition zone diameters of all isolates are provided as mean ± SD in Supplementary Table S2. Multidrug resistance (MDR) was defined as resistance to at least one antimicrobial agent in three or more antimicrobial classes [31]. The multiple antibiotic resistance (MAR) index was determined as the ratio of antimicrobial agents to which each isolate was found to be resistant divided by the total number of antimicrobial agents tested [6,7,23,31,37,38,39]. All antimicrobial susceptibility assays were performed in triplicate, and the mean values were used for final interpretation.

### 2.6. Quantification of Biofilm Formation by Crystal Violet Assay 

The biofilm-forming ability of bacterial isolates was evaluated using the crystal violet microtiter plate assay according to previously described methods [31,40]. *A. veronii* isolates were cultured overnight in tryptic soy broth (TSB) and then adjusted to approximately 1 × 10^8^ CFU/mL. A 200 µL aliquot of each standardized bacterial suspension was transferred into sterile 96-well flat-bottom microplates. Each isolate was tested in three independent microtiter plate experiments, with three technical wells per isolate in each experiment. Wells containing uninoculated TSB served as negative controls.

The microplates were incubated under static conditions at 32 °C for 24 h. Planktonic cells were removed after incubation, and wells were washed three times with sterile PBS (1×; pH 7.4) to remove non-adherent cells. The remaining attached biofilm biomass was fixed with methanol and stained with 0.1% (*w*/*v*) crystal violet solution for 1 h at room temperature. Excessive stain was removed by washing with sterile distilled water, followed by air drying. The bound crystal violet was dissolved in 95% ethanol, and absorbance was measured at OD_595_ using a microplate reader (Bambi Technology, Shanghai, China).

For each independent experiment, the mean OD_595_ was calculated from the three technical wells for each independent experiment. Final biofilm data were expressed as mean OD_595_ ± SD from three independent experiments. Biofilm production was classified according to the optical density cut-off (ODc) method [31], where ODc was calculated as the mean OD595 value of the negative control plus three times its standard deviation. Isolates were classified as non-biofilm producers (OD ≤ ODc), weak biofilm producers (ODc < OD ≤ 2ODc), moderate biofilm producers (2ODc < OD ≤ 4ODc), and strong biofilm producers (OD > 4ODc) [31,40]. The crystal violet assay, performed under clearly defined experimental conditions, provides a quantitative estimation of total biofilm biomass and is widely used for comparative evaluation of biofilm-forming capacity, with detailed reporting of assay conditions being essential for appropriate interpretation and reproducibility of results [41,42].

### 2.7. Statistical Analysis

All quantitative data are presented as mean ± standard deviation (SD) from three replicates. Statistical analysis and graphical presentation were performed using GraphPad Prism 11.0 (GraphPad Software, San Diego, CA, USA). Descriptive statistics were used to analyze the antimicrobial susceptibility profiles, multiple antibiotic resistance (MAR) indices, and biofilm-forming ability of *A. veronii* isolates. Quantitative measurements were summarized as mean ± SD, while categorical observations were presented according to their respective classifications.

## 3. Results

### 3.1. Bacterial Isolation and Phenotypic Characterization

**Figure 2:**
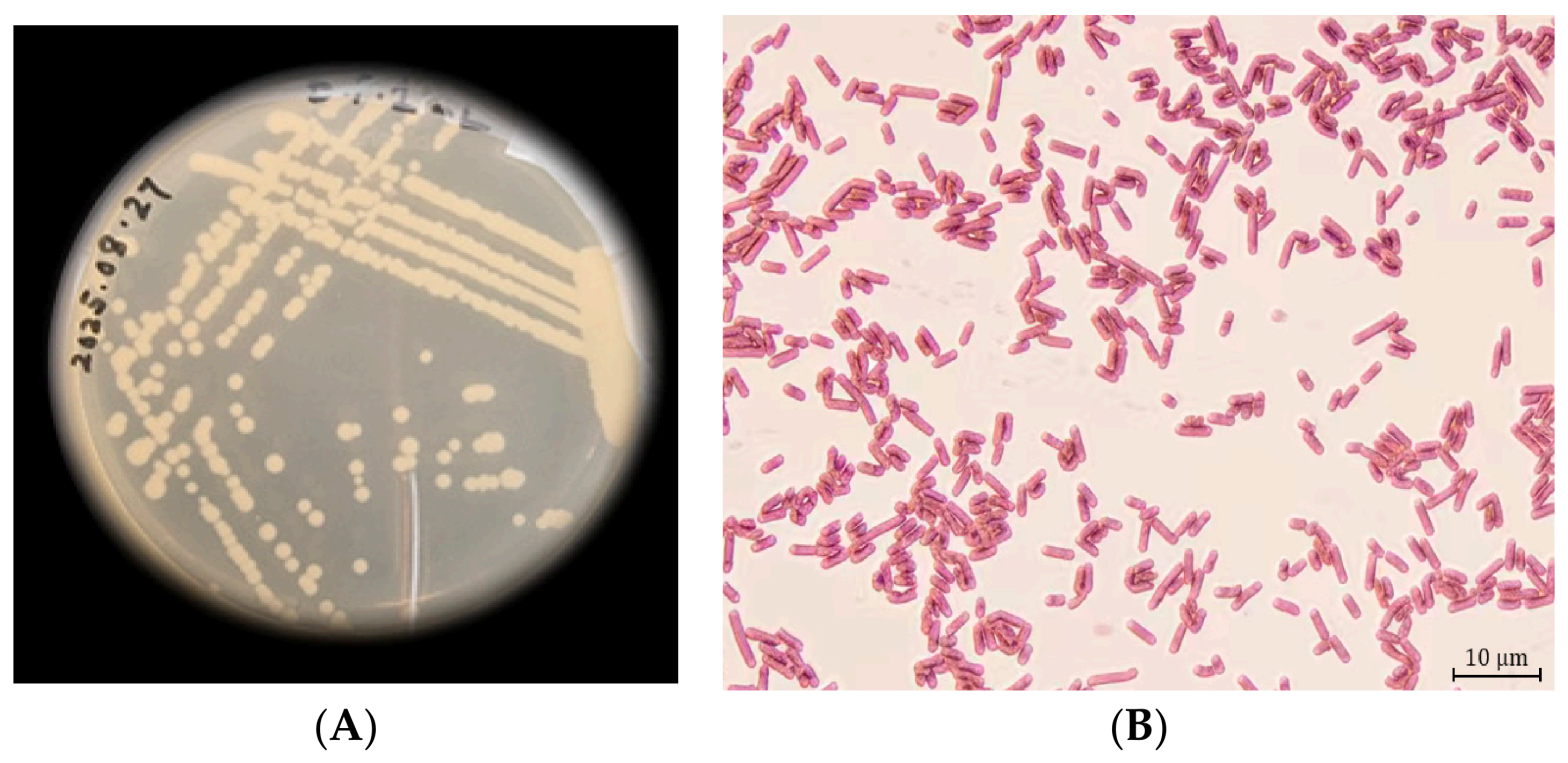
Morphological characteristics of *Aeromonas veronii* isolate DMNZ06 (representative isolate) recovered from *Diptychus maculatus*. (**A**) Colony morphology on tryptic soy agar after incubation; (**B**) Gram-negative rod-shaped cells observed under light microscopy at 1000× magnification with immersion oil.

A total of eight bacterial isolates were recovered from eight individual *D. maculatus* (one isolate per fish) and were designated DMNZ01-DMNZ08. The isolates were obtained from different sampled anatomical sites, including eye (DMNZ01), intestine (DMNZ02), dorsal fin (DMNZ03), body kidney (DMNZ04), skin (DMNZ05), gill (DMNZ06), gonad (DMNZ07), and spleen (DMNZ08) (Supplementary Table S1). No bacterial isolates were recovered from the anus and liver samples of the remaining two fish under the applied isolation conditions. All isolates exhibited comparable colony morphology on TSA, including creamy-white coloration, smooth surface, circular shape, entire margins, and convex elevation (Figure 2A). All isolates were Gram-negative, rod-shaped bacterial cells by Gram staining and microscopic examination (Figure 2B).

All isolates showed identical biochemical reaction profiles under the tested conditions using a commercial biochemical identification system (Table 1). All were positive for oxidase, catalase, citrate utilization, indole, methyl red (MR), Voges–Proskauer (VP), lysine decarboxylase, and ornithine decarboxylase. A motility test showed that all isolates were motile, as indicated by diffuse growth in semi-solid medium. Negative reactions were observed for MUG and carbohydrate utilization tests, including lactose, raffinose, xylose, cellobiose, and sorbitol. These phenotypic characteristics were consistent with the preliminary identification of the isolates as members of the genus *Aeromonas* [17,21,26], and further molecular analyses were subsequently performed for species-level identification.

### 3.2. Molecular Identification Based on 16S rRNA Gene Sequencing and Confirmatory gyrB Analysis

**Figure 3:**
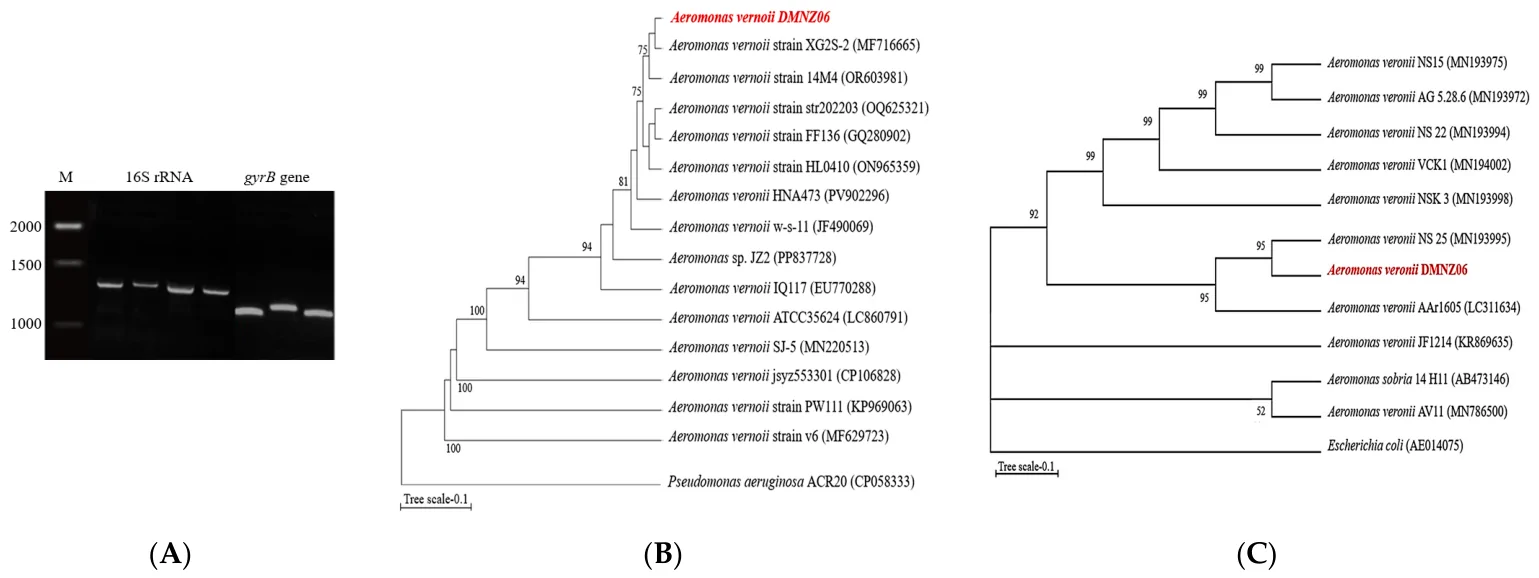
Molecular identification of *Aeromonas veronii* isolates recovered from *Diptychus maculatus*. (**A**) Agarose gel electrophoresis showing PCR amplification products. Lane M represents the DNA marker; 16S rRNA gene amplification products (~1400 bp) and *gyrB* gene amplification products (~1100 bp) from representative isolate DMNZ06. (**B**,**C**) Neighbor-Joining phylogenetic trees based on 16S rRNA and *gyrB* sequences, respectively, with 1000 bootstrap replicates; scale bars are indicated.

PCR amplification of the 16S rRNA gene from the recovered bacterial isolates generated amplicons of approximately 1400 bp, corresponding to the expected size of bacterial 16S rRNA gene fragments [17] (Figure 3A). High-quality sequences were obtained and compared with the reference sequences available in the NCBI GenBank database by BLASTn analysis. The obtained sequences of all isolates showed 99.8–100% similarity, 100% query coverage, and an E-value of 0.0 with reference sequences of *A. veronii*, supporting their preliminary identification as *A. veronii*. Comparative analysis among the recovered isolates showed highly similar 16S rRNA gene sequences (99.8–100% similarity). Therefore, representative isolate DMNZ06 was selected for further *gyrB*-based confirmation. Phylogenetic analysis, based on 16S rRNA gene sequences, was carried out using the Neighbor-Joining method (1000 bootstrap replications). The representative isolate DMNZ06 clustered within the *A. veronii* lineage and was distinct from closely related *Aeromonas* species, with 75% bootstrap support (Figure 3B).

The *gyrB* gene of the representative isolate DMNZ06 was subsequently amplified and sequenced for additional species-level resolution. The *gyrB* sequence obtained was approximately 1100 bp in length and showed 99.11% similarity with 100% query coverage and an E-value of 0.0 with the reference *A. veronii* sequences in GenBank [17]. The phylogenetic analysis based on *gyrB* sequences demonstrated that DMNZ06 clustered within the *A. veronii* clade with strong bootstrap support (95%), showing a close relationship with previously reported *A. veronii strains* (Figure 3C). Collectively, 16S rRNA gene sequencing of the recovered isolates, together with confirmatory *gyrB* analysis of representative isolate DMNZ06, supported the identification of the recovered isolates as *A. veronii*.

### 3.3. Antimicrobial Susceptibility Profile

All recovered *A. veronii* isolates exhibited highly concordant antimicrobial susceptibility profiles across the tested agents (Table 2). The isolates were susceptible to enrofloxacin, cefotaxime, ceftriaxone, and florfenicol, whereas intermediate susceptibility was observed for ciprofloxacin, ofloxacin, doxycycline, oxytetracycline, and trimethoprim–sulfamethoxazole. Resistance was noted against norfloxacin, neomycin, penicillin, amoxicillin, tetracycline, and erythromycin. The inhibition zone diameters from the disc diffusion assay are reported as mean ± SD in Supplementary Table S2. The reference strain *A. veronii* BNCC138468 showed a distinct susceptibility pattern for some of the tested agents (enrofloxacin, ciprofloxacin, tetracycline, and erythromycin), providing a comparative reference for interpretation of the susceptibility profiles under the same experimental conditions and interpretation criteria applied to the recovered isolates.

The observed resistance phenotype involved multiple antimicrobial classes, including fluoroquinolones, aminoglycosides, β-lactams, tetracyclines, and macrolides, while cephalosporins and florfenicol remained effective under the tested conditions. Based on the defined criteria, the recovered isolates exhibited a multidrug-resistant (MDR) phenotype, showing resistance to at least one antimicrobial agent belonging to three or more antimicrobial classes. Since all isolates displayed the same categorical resistance pattern, the conventional MAR index, calculated using all resistant antimicrobial agents, was 0.40 (6 resistant agents/15 tested agents) for each isolate (mean MAR index = 0.40 ± 0.00). The adjusted MAR index, excluding penicillin and amoxicillin due to their recognized intrinsic β-lactam resistance, was 0.31 (4 resistant agents/13 tested agents). Similar antimicrobial susceptibility profiles observed among the recovered isolates indicate a shared resistance pattern under the tested conditions and highlight the importance of monitoring antimicrobial resistance in conservation-raised fish stocks.

### 3.4. Biofilm-Forming Ability of the Isolates

The biofilm-forming ability of the recovered *A. veronii* isolates was evaluated using the crystal violet microtiter plate assay. The quantitative assessment based on OD_595_ measurements showed that all isolates produced detectable levels of biofilm biomass under the tested conditions, with OD_595_ values ranging from 0.18 to 0.38, compared with the negative control value of 0.11 ± 0.01 (Table 3). The OD cut-off value (ODc), calculated as the mean OD_595_ value of the negative control plus three times its standard deviation, was 0.14. Based on the ODc classification criteria, DMNZ01 (0.18 ± 0.02), DMNZ02 (0.25 ± 0.02), DMNZ04 (0.19 ± 0.01), and DMNZ07 (0.20 ± 0.01) were categorized as weak biofilm producers, whereas DMNZ03 (0.30 ± 0.02), DMNZ05 (0.32 ± 0.02), DMNZ06 (0.38 ± 0.02), and DMNZ08 (0.30 ± 0.02) were classified as moderate biofilm producers. Among the tested isolates, DMNZ06 exhibited the highest OD_595_ value, indicating the greatest biofilm biomass under the assay conditions.

Overall, the recovered isolates displayed variation in biofilm-forming capacity under standardized in vitro conditions. The findings indicate differences in biofilm biomass production among the isolates. However, the observed categories represent phenotypic responses under the specific assay conditions applied and should not be interpreted as direct evidence of ecological persistence or enhanced survival capacity in natural environments.

## 4. Discussion

Freshwater fish inhabiting high-altitude cold-water systems represent unique biological systems, characterized by low temperatures, seasonal environmental fluctuations, and distinct ecological conditions, where associated bacterial taxa may influence host health and interactions with the surrounding environment [2,5]. However, information on microorganisms associated with many protected freshwater fish species remains limited, particularly regarding bacterial taxa with potential relevance to fish health. Members of the genus *Aeromonas* are widely distributed in aquatic environments and include several opportunistic pathogens associated with disease conditions in fish and other aquatic organisms [7]. The present study provides the first report and integrated characterization of *A. veronii* associated with *D. maculatus*, a second-class protected fish species inhabiting freshwater ecosystems of Xinjiang, China [4]. The findings provide baseline information regarding the occurrence, prevalence, antimicrobial susceptibility profiles and biofilm-associated properties of *A. veronii* isolates from this conservation-managed fish group.

### 4.1. Detection of Aeromonas veronii in a Protected Cold-Water Fish

In this study, *A. veronii* was recovered from various tissues of selected *D. maculatus* individuals, including eye, intestine, dorsal fin, body kidney, gill, skin, gonad, and spleen. Most previous research on *D. maculatus* has primarily focused on taxonomy, ecology, reproductive biology, and conservation status, whereas information regarding culturable bacteria associated with this species remains scarce. Therefore, characterizing potentially significant bacterial taxa associated with this protected fish species represents an important component of conservation-oriented health assessment [6,8]. The recovery of *A. veronii* isolates from multiple anatomical sites among sampled fish indicates its association with these sampled tissues. However, these findings do not establish tissue-specific colonization or infection. Similar approaches have been applied in aquatic microbiology studies to evaluate the occurrence of potentially important bacterial taxa among different tissues of fish hosts [14,43]. However, tissue recovery alone cannot differentiate between active infection, transient association, and colonization. Thus, the presence of *A. veronii* should be interpreted as a bacterial association among the sampled fish rather than direct evidence of tissue invasion or disease causation.

Potential contamination was minimized through surface disinfection, individual tissue processing, and the use of sterilized instruments between successive tissue collections. However, no instrument or dissection-surface swab controls were performed. Although uninoculated TSA/PBS controls were included, they could not rule out possible tissue-to-tissue carryover during dissection. Therefore, the recovery of *A. veronii* from internal tissues should be interpreted with caution. Because bacterial counts were not quantified, the present study provides evidence of bacterial recovery under the applied culture conditions but does not determine the quantitative bacterial load within individual tissues.

The bacterial recovery procedure was conducted under standardized laboratory conditions commonly applied for mesophilic *Aeromonas* spp. isolation, which may differ from the lower temperature experienced by *D. maculatus* in its natural environment. Accordingly, the recovered isolates provide phenotypic information on culturable bacterial populations associated with the sampled fish, while interpretation should consider the influence of laboratory cultivation conditions. *A. veronii* is known to be an opportunistic aquatic pathogen and has been reported from many freshwater fish species, where it has been linked to disease outbreaks under favorable conditions [13,37]. Its pathogenicity is considered highly context-dependent and influenced by host physiological state, environmental factors, and strain-specific genetic factors [44]. Therefore, the ecological and health significance of detecting *A. veronii* in protected fish populations requires further evaluation through integrated approaches combining microbial characterization, environmental assessment, and host–microbe interaction studies.

In the present study, bacterial isolation was performed following observations of mortality events at the breeding facility. However, the study was not designed to establish a causal relationship between *A. veronii* and mortality. Additional investigations involving environmental parameters, histopathological evaluation, bacterial load quantification, virulence-associated gene screening, and experimental infection models are required to determine the pathogenic role of these isolates in *D. maculatus*. Nevertheless, the detection of a recognized opportunistic pathogen in a protected fish species maintained under conservation management conditions indicates the need for continuous microbial surveillance. Routine monitoring of bacterial occurrence and phenotypic characteristics may support early identification of potentially important microbial changes and contribute to improved health management strategies in conservation breeding programs. Considering the documented pathogenic potential of *A. veronii* in freshwater aquaculture systems, the detection of this bacterium in *D. maculatus* provides valuable baseline information for future studies of host–microbe interactions in this species.

### 4.2. Phenotypic and Molecular Confirmation of the Isolates

The recovered isolates exhibited phenotypic characteristics consistent with the genus *Aeromonas*, including rod-shaped morphology, Gram-negative staining, motility, and positive oxidase and catalase reactions. Biochemical profiling showed a high degree of phenotypic concordance among the recovered isolates. However, biochemical identification alone is insufficient to provide adequate resolution for discrimination among closely related *Aeromonas* species, particularly those belonging to the *A. hydrophila* complex, for which overlapping biochemical characteristics are frequently observed [45,46,47]. To overcome the limitations associated with phenotypic identification, the present study integrated biochemical characterization and molecular approaches. The 16S rRNA gene served as the primary molecular marker for taxonomic identification and phylogenetic placement. The 16S rRNA gene sequence analysis of the recovered isolates showed high similarity with reference *A. veronii* sequences and supported their assignment to *A. veronii*.

Although 16S rRNA sequencing remains one of the most widely used bacterial identification methods, its discriminatory power may be limited among closely related taxa because of sequence conservation. Therefore, sequencing of the *gyrB* gene was additionally performed for the representative isolate DMNZ06 to provide improved species-level resolution [48]. Housekeeping genes such as *gyrB*, *rpoB*, and *rpoD* generally show greater sequence variability than 16S rRNA and have been widely used for differentiation of closely related *Aeromonas* species [48,49,50]. The *gyrB* analysis provided additional species-level confirmation for DMNZ06, while the consistent phenotypic characteristics and 16S rRNA sequence-based identification supported the assignment of the remaining isolates as *A. veronii*. However, the molecular analyses were not designed to assess strain-level genetic relatedness among the recovered isolates. Accurate taxonomic identification is particularly important in ecological and wildlife microbiology studies because closely related bacterial species may exhibit substantial differences in virulence potential, antimicrobial resistance, and ecological behavior.

### 4.3. Antimicrobial Susceptibility Pattern and Its Significance

The antimicrobial susceptibility analysis revealed a similar resistance phenotype among the eight recovered *A. veronii* isolates. All isolates showed susceptibility to enrofloxacin, cefotaxime, ceftriaxone, and florfenicol, resistance to norfloxacin, neomycin, penicillin, amoxicillin, tetracycline, and erythromycin, and intermediate responses to several fluoroquinolones and tetracycline derivatives [7,51]. The reference strain *A. veronii* BNCC138468 exhibited different susceptibility responses to several antimicrobial agents, providing a comparative reference for interpretation of the susceptibility profiles. The observed resistance pattern involved multiple antimicrobial classes, including β-lactams, aminoglycosides, tetracyclines, and macrolides. Resistance to penicillin and amoxicillin is consistent with previously described intrinsic resistance mechanisms in *Aeromonas* species, which are commonly associated with chromosomally encoded β-lactamases [37,51,52]. Therefore, resistance to these agents should be interpreted cautiously when evaluating multidrug resistance patterns.

Reduced susceptibility to erythromycin and tetracycline derivatives has also been reported among other bacterial species, although comparison needs to consider differences in bacterial species, strains, antimicrobial exposure (intrinsic and acquired resistance), and testing conditions [51].

It is important to note that the susceptibility interpretation of aquatic *Aeromonas* isolates remains challenging, as many antimicrobial agents and ecological variants of *A. veronii* do not have standardized criteria. Susceptibility categories assigned in the present study were based on published *Aeromonas*-related interpretation for the antimicrobial agents and disk concentrations tested [21,23,35,36]. Although incubation conditions may affect inhibition zone measurements, all the isolates and the reference strain were tested under the same experimental conditions, allowing for comparative assessment of their relative phenotypic susceptibility profiles.

Although all isolates fulfilled the phenotypic criteria for multidrug resistance based on resistance to antimicrobial agents from three or more classes, the similar susceptibility profiles indicate a shared phenotypic pattern rather than evidence of identical resistance mechanisms or genetic relatedness. The observed MAR index value was 0.40 for all isolates based on the complete antimicrobial panel. Following the exclusion of penicillin and amoxicillin due to their recognized intrinsic β-lactam resistance characteristics in *Aeromonas*, the adjusted MAR index was 0.31.

However, this value should be interpreted cautiously because resistance to some tested agents, particularly β-lactams, may involve intrinsic resistance mechanisms characteristic of *Aeromonas* species [7,19,21,39]. The high concordance of antimicrobial susceptibility and biochemical profiles suggests a possible closely related strain background. However, genetic relatedness and clonality were not evaluated in the present study. Species-level identification and phenotypic characterization of recovered isolates were the focus of the study, whereas strain-level typing was beyond its scope. Therefore, the observed phenotypic uniformity should not be interpreted as evidence of genetically independent strains or generalized as a uniform characteristic of *A. veronii* populations.

In aquatic production systems, antimicrobial exposure associated with disease prevention or treatment practices has been recognized as an important selective pressure contributing to the emergence and persistence of resistant bacteria. Furthermore, intrinsic resistance mechanisms and environmental dissemination routes may also affect the pattern of resistance within aquatic *Aeromonas* populations. Previous reports, such as isolation of *A. veronii* from largemouth bass in China, have demonstrated inter-strain diversity in both pathogenicity and drug resistance, supporting the notion that *A. veronii* cannot be considered a homogeneous species solely based on susceptibility profiles [14,29,37,53]. The observed susceptibility to the third-generation cephalosporines aligns with reports of variable susceptibility patterns, depending on strain and environmental context. Florfenicol remained effective, consistent with its frequent use in aquaculture, although emerging resistance has been reported in some systems [51,54,55,56]. However, the relationship between these resistance patterns and previous antimicrobial exposure cannot be determined because detailed antimicrobial usage records at the breeding facility were unavailable.

The presence of multidrug-resistant *Aeromonas* in aquatic environments is a growing concern because aquatic systems facilitate the persistence and dissemination of resistant bacteria [57,58]. From a conservation perspective, baseline antimicrobial susceptibility can provide valuable reference data for future health management, particularly in captive breeding and release programs. Future research incorporating antimicrobial resistance gene analysis and environmental surveillance will provide a better understanding of the ecological significance and mechanisms of antimicrobial resistance in the *A. veronii* population.

### 4.4. Biofilm Formation and Biological Interpretation

Biofilm formation is a significant adaptive trait that contributes to bacterial attachment, persistence, and survival under changing environmental conditions [59,60]. Biofilm-associated growth can improve the ability of aquatic bacteria to persist on biotic and abiotic surfaces and to survive over extended periods compared with planktonic bacteria. However, these properties are highly dependent on environmental and experimental factors. In the present study, all recovered *A. veronii* isolates showed measurable biofilm-associated biomass under the applied crystal violet assay conditions.

According to the ODc-based classification method, the isolates exhibited weak to moderate biofilm formation capacity, with variation among individual isolates. The highest OD_595_ value was observed for DMNZ06, while the biomass production was comparatively lower in the other isolates. Similar biofilm-forming abilities have been reported among *Aeromonas* species, supporting the importance of surface-associated growth as a common bacterial survival strategy [61,62]. Biofilm formation and virulence in *A. veronii* are influenced by multiple regulatory systems associated with bacterial adaptation, motility, and chemotaxis, suggesting that this phenotype may contribute to ecological fitness and environmental adaptation [7,63].

Additionally, the similarity in the phenotypic characteristics of the recovered isolates may reflect a similar or closely related strain background. However, the present study did not assess strain-level relatedness. Consequently, the observed phenotypes should be evaluated as characteristic of the recovered set of isolates under the applied assay conditions, rather than as evidence of strain-independent biofilm traits.

However, biofilm formation is highly dependent on the experimental conditions such as temperature, nutrient availability, duration of incubation and surface characteristics. Thus, the categories of biofilm observed represent in vitro phenotypic responses to the specific assay conditions and should not be interpreted as direct evidence of enhanced persistence or pathogenicity within the natural habitat of *D. maculatus*. Biofilm formation may contribute to bacterial survival and host interaction potential, but additional investigations involving environmental conditions, genomic analysis of biofilm-related genes, and host response studies are needed to determine its ecological significance [40].

As a baseline characterization study, the present work provides initial insights into the occurrence of *A. veronii* associated with *D. maculatus,* as well as its phenotypic properties. Several limitations should be considered when interpreting these findings. The study involved a single sampling event and recovery of a limited number of isolates. Thus, further temporal and spatial variation needs to be evaluated.

Although basic information regarding the sampling location and fish management conditions was recorded, environmental bacterial reservoirs, such as water- and sediment-associated microbial communities, were not investigated. In addition, concurrent measurements of habitat temperature and water physicochemical parameters were not performed during sampling. Although the high-altitude, cold-water habitat of D. maculatus [5] and the temperature conditions maintained at the breeding station provide relevant ecological context, the lack of site-specific environmental measurements limits assessment of the relationship between the occurrence of *A. veronii* and the environmental conditions prevailing at the sampling site. Furthermore, molecular analyses focused on taxonomic identification, while virulence-associated genes, antimicrobial resistance determinants, and whole-genome characteristics were not examined. Additionally, histopathological and experimental infection studies were not performed, and clinical observations were not systematically scored for individual fish. Therefore, definitive conclusions cannot be drawn regarding the pathogenic role of the recovered isolates. Future research combining longitudinal sampling, environmental microbiology, genomic approaches, and host response analysis will provide a deeper understanding of *A. veronii* ecology and its relevance to conservation-oriented health management of protected freshwater fish.

## 5. Conclusions

This study provides the first baseline report and integrated characterization of *A. veronii* associated with *D. maculatus* from a high-altitude freshwater ecosystem in Xinjiang, China. Phenotypic characterization and 16S rRNA gene sequencing supported the assignment of the recovered isolates to *A. veronii*, while additional *gyrB* analysis provided species-level confirmation for the representative isolate DMNZ06. The recovered isolates exhibited a consistent antimicrobial susceptibility profile, including resistance to multiple antimicrobial classes, and showed weak to moderate biofilm-associated biomass production under the specific in vitro assay conditions applied. The recovery of *A. veronii* from multiple tissues of a protected freshwater fish species provides reference information regarding host-associated bacterial occurrence and phenotypic characteristics. Although this study does not establish disease causation, virulence mechanisms, or the genetic basis underlying antimicrobial resistance and biofilm-associated traits, it highlights the value of microbial characterization in conservation-oriented fish health studies. Future investigations incorporating genomic analyses, virulence assessment, and broader ecological sampling will further elucidate the biological importance of *A. veronii* in *D. maculatus* populations.

## Figures and Tables

**Table 1:** Biochemical and physiological characteristics of *Aeromonas veronii* isolates recovered from *D. maculatus.*

Biochemical Test	DMNZ 01	DMNZ 02	DMNZ 03	DMNZ 04	DMNZ 05	DMNZ 06	DMNZ 07	DMNZ 08	*A. veronii* BNCC138468
Oxidase	+	+	+	+	+	+	+	+	+
Catalase	+	+	+	+	+	+	+	+	+
Citrate utilization	+	+	+	+	+	+	+	+	+
Indole production	+	+	+	+	+	+	+	+	+
Methyl Red (MR)	+	+	+	+	+	+	+	+	−
Voges–Proskauer (VP)	+	+	+	+	+	+	+	+	+
Raffinose utilization	−	−	−	−	−	−	−	−	−
Cellobiose utilization	−	−	−	−	−	−	−	−	+
Sorbitol utilization	−	−	−	−	−	−	−	−	−
Lactose	−	−	−	−	−	−	−	−	−
Xylose	−	−	−	−	−	−	−	−	
Lysine decarboxylase	+	+	+	+	+	+	+	+	+
Ornithine decarboxylase	+	+	+	+	+	+	+	+	+
MUG	−	−	−	−	−	−	−	−	−
Motility	+	+	+	+	+	+	+	+	+

+ (Positive), − (Negative).

**Table 2:** Antimicrobial susceptibility profile of *A. veronii* isolates recovered from *D. maculatus* (S: susceptible; I: intermediate; R: resistant).

Antibiotic Class	Antibiotic Disc Content		Interpretation	
			DMNZ01–DMNZ08	BNCC 138468
Fluoroquinolones	Enrofloxacin	5 µg	S	S
	Ciprofloxacin	5 µg	I	S
	Norfloxacin	10 µg	R	R
	Ofloxacin	5 µg	I	I
Aminoglycosides	Neomycin	30 µg	R	R
β-lactams	Penicillin	10 IU	R	R
	Amoxicillin	10 µg	R	R
Cephalosporins	Cefotaxime	30 µg	S	S
	Ceftriaxone	30 µg	S	S
Tetracyclines	Tetracycline	30 µg	R	I
	Doxycycline	30 µg	I	S
	Oxytetracycline	30 µg	I	S
Folate inhibitors	Trimethoprim-sulfamethoxazole	1.25/23.75 µg	I	S
Macrolides	Erythromycin	15 µg	R	I
Amphenicols	Florfenicol	30 µg	S	S

**Table 3:** Quantitative biofilm formation of *Aeromonas veronii* isolates determined by the crystal violet microtiter plate assay ^1^.

	Control	DMNZ01	DMNZ02	DMNZ03	DMNZ04	DMNZ05	DMNZ06	DMNZ07	DMNZ08
Mean OD_595_	0.11 ± 0.01	0.18 ± 0.02	0.25 ± 0.02	0.30 ± 0.02	0.19 ± 0.01	0.32 ± 0.02	0.38 ± 0.02	0.20 ± 0.01	0.30 ± 0.02
Degree of Biofilm	-	Weak	Weak	Moderate	Weak	Moderate	Moderate	Weak	Moderate

^1^ Values represent mean OD_595_ ± SD from three independent experiments. Biofilm categories were assigned using the ODc method (ODc = 0.14): non-producer (≤0.14), weak (0.14–0.28), moderate (0.28–0.56), and strong (>0.56).

## Data Availability

The data supporting the findings of this study are available from the corresponding author upon reasonable request.

## Supplementary Materials

The following supporting information can be downloaded at https://www.mdpi.com/article/10.3390/ani16172787/s1, Table S1. Metadata and traceability information of *Aeromonas veronii* isolates recovered from individual *Diptychus maculatus* specimens; Table S2. Raw inhibition zone diameters (mm) of *Aeromonas veronii* isolates (DMNZ01–DMNZ08) and reference strain obtained from triplicate antimicrobial susceptibility assays.

## References

[1] Reid A.J., Carlson A.K., Creed I.F., Eliason E.J., Gell P.A., Johnson P.T., Kidd K.A., MacCormack T.J., Olden J.D., Ormerod S.J. (2019). Emerging threats and persistent conservation challenges for freshwater biodiversity. Biol. Rev..

[2] Samsing F., Barnes A.C. (2024). The rise of the opportunists: What are the drivers of the increase in infectious diseases caused by environmental and commensal bacteria?. Rev. Aquac..

[3] Hu L., Han A., Song Y., Yang L., Serekbol G., Liu J., Huo B., Ren D., Wang C., Chen S. (2025). Threatened fishes of the world: *Diptychus maculatus* and *Aspiorhynchus laticeps*. Isr. J. Aquac. Bamidgeh.

[4] Li G., Tang Y., Zhang R., Zhao K. (2016). Phylogeography of *Diptychus maculatus* (Cyprinidae) endemic to the northern margin of the QTP and Tien Shan region. BMC Evol. Biol..

[5] Nie Z., Hao H., Zhao H., Zhao N., Li L., Qiang Z., Hamid S.M., Wei J. (2025). Effects of Water Temperature, Light Intensities and Photoperiod on the Survival and Growth of Juvenile *Schizothorax irregularis* and *Diptychus maculates*. Fishes.

[6] Trevelline B.K., Fontaine S.S., Hartup B.K., Kohl K.D. (2019). Conservation biology needs a microbial renaissance: A call for the consideration of host-associated microbiota in wildlife management practices. Proc. R. Soc. B Biol. Sci..

[7] Tao L.T., Gao D.P., Liu Y.L., Sun W.W., Shan X.F. (2026). The Genus *Aeromonas* in Aquaculture: A Comprehensive Review of Prevalence, Virulence, and Antibiotic Resistance With an Emphasis on Key Pathogenic Species. Rev. Aquac..

[8] Xu X., Fu H., Wan G., Huang J., Zhou Z., Rao Y., Liu L., Wen C. (2022). Prevalence and genetic diversity of *Aeromonas veronii* isolated from aquaculture systems in the Poyang Lake area, China. Front. Microbiol..

[9] Medina-Félix D., Garibay-Valdez E., Vargas-Albores F., Martínez-Porchas M. (2023). Fish disease and intestinal microbiota: A close and indivisible relationship. Rev. Aquac..

[10] Kanika N.H., Liaqat N., Chen H., Ke J., Lu G., Wang J., Wang C. (2025). Fish gut microbiome and its application in aquaculture and biological conservation. Front. Microbiol..

[11] Minich J.J., Härer A., Vechinski J., Frable B.W., Skelton Z.R., Kunselman E., Shane M.A., Perry D.S., Gonzalez A., McDonald D. (2022). Host biology, ecology and the environment influence microbial biomass and diversity in 101 marine fish species. Nat. Commun..

[12] Ibrahim G.A., Mabrok M., Alfifi K.J., Alatawy M., Al-otaibi A.S., Alenzi A.M., Rahman A.N.A., El-Malt R.M., Ibrahim S.A., El-Tarabili R.M. (2024). Pathogenicity, resistance patterns, virulence traits, and resistance genes of re-emerging extensively drug-resistant (XDR) *Aeromonas veronii* in *Oreochromis niloticus*. Aquac. Int..

[13] Maia J.C.D.S., Silva G.A.D.A., Cunha L.S.D.B., Gouveia G.V., Góes-Neto A., Brenig B., Araújo F.A., Aburjaile F., Ramos R.T.J., Soares S.C. (2023). Genomic characterization of Aeromonas veronii provides insights into taxonomic assignment and reveals widespread virulence and resistance genes throughout the world. Antibiotics.

[14] Chen Q., Xing Y., Lei Y., Tong G., Lin X., He P., Tang S., Zheng F., Zeng H., Wei X. (2024). Genetic diversity, antibiotic resistance, and pathogenicity of *Aeromonas veronii* isolated from farmed largemouth bass (*Micropterus salmoides*) in the main aquaculture regions of China. Aquaculture.

[15] Sekizuka T., Tanaka R., Hashino M., Yatsu K., Kuroda M. (2022). Comprehensive genome and plasmidome analysis of antimicrobial resistant bacteria in wastewater treatment plant effluent of Tokyo. Antibiotics.

[16] Judan Cruz K.G., Takumi O., Bongulto K.A., Gandalera E.E., Kagia N., Watanabe K. (2024). Natural compound-induced downregulation of antimicrobial resistance and biofilm-linked genes in wastewater *Aeromonas* species. Front. Cell. Infect. Microbiol..

[17] Chen F., Sun J., Han Z., Yang X., Xian J.A., Lv A., Hu X., Shi H. (2019). Isolation, identification and characteristics of *Aeromonas veronii* from diseased crucian carp (*Carassius auratus gibelio*). Front. Microbiol..

[18] Zhang J., Qiao D., Wang H., Zhao X., Jiang X., Zhu L., Zhang J., Li L., Kong X., Pei C. (2025). Mixed infection in common carp (*Cyprinus carpio*) caused by *Aeromonas veronii*, *Aeromonas hydrophila*, *Plesiomonas shigelloides*, and *Citrobacter freundii*. Animals.

[19] Tekedar H.C., Arick M.A., Hsu C.Y., Thrash A., Blom J., Lawrence M.L., Abdelhamed H. (2020). Identification of antimicrobial resistance determinants in *Aeromonas veronii* strain MS-17-88 recovered from channel catfish (*Ictalurus punctatus*). Front. Cell. Infect. Microbiol..

[20] Mallik S.K., Joshi N., Shahi N., Kala K., Singh S., Giri A.K., Pant K., Chandra S. (2020). Characterization and pathogenicity of *Aeromonas veronii* associated with mortality in cage farmed grass carp, *Ctenopharyngodon idella* (Valenciennes, 1844) from the Central Himalayan region of India. Antonie Leeuwenhoek.

[21] Behera B.K., Parda S.N., Kumar V., Swain H.S., Parida P.K., Bisai K., Dhar S., Das B.K. (2023). Aeromonas veronii is a lethal pathogen isolated from gut of infected Labeo rohita: Molecular insight to understand the bacterial virulence and its induced host immunity. Pathogens.

[22] Lian H.M., Li S.W., Zhang H., Lu Z.H., Lu T.Y. (2015). Distribution and antimicrobial susceptibility analysis of common pathogenic bacteria isolated from cold-water fish in three north areas of China. J. Dalian Fish. Univ..

[23] Ayoub H.F., Tohamy E.Y., Mohamed S.S. (2021). Isolation, Identification and Antimicrobial profile of *Aeromonas* spp., *Pseudomonas* spp. and *Vibrio* spp. from the Nile Tilapia, *Oreochromis niloticus* in fish farms. Egypt. J. Aquat. Biol. Fish..

[24] Hu X., Xiao Z., Li B., Xue M., Jiang N., Fan Y., Chen P., Qi F., Kong X., Zhou Y. (2023). Isolation, identification, and characterization of *Aeromonas veronii* from Chinese soft-shelled turtle (*Trionyx sinensis*). Microorganisms.

[25] Tekedar H.C., Kumru S., Blom J., Perkins A.D., Griffin M.J., Abdelhamed H., Karsi A., Lawrence M.L. (2019). Comparative genomics of *Aeromonas veronii*: Identification of a pathotype impacting aquaculture globally. PLoS ONE.

[26] Fadel A., Mahmoud M.A., Abdelsalam M., Eissa E.S.H., Sherif A.H. (2025). *Aeromonas veronii* infection in cultured *Oreochromis niloticus*: Prevalence, molecular and histopathological characterization correlated to water physicochemical characteristics, with the protective autochthonous probiotic. Aquac. Int..

[27] Chen H., Zhao Y., Chen K., Wei Y., Luo H., Li Y., Liu F., Zhu Z., Hu W., Luo D. (2022). Isolation, identification, and investigation of pathogenic bacteria from common carp (*Cyprinus carpio*) naturally infected with *Plesiomonas shigelloides*. Front. Immunol..

[28] Sun J., Zhang X., Gao X., Jiang Q., Wen Y., Lin L. (2016). Characterization of virulence properties of *Aeromonas veronii* isolated from diseased Gibel Carp (*Carassius gibelio*). Int. J. Mol. Sci..

[29] Wang B., Mao C., Feng J., Li Y., Hu J., Jiang B., Gu Q., Su Y. (2021). A First Report of *Aeromonas veronii* Infection of the Sea Bass, *Lateolabrax maculatus* in China. Front. Vet. Sci..

[30] Woo S.J., Kim M.S., Jeong M.G., Do M.Y., Hwang S.D., Kim W.J. (2022). Establishment of epidemiological cut-off values and the distribution of resistance genes in *Aeromonas hydrophila* and *Aeromonas veronii* isolated from aquatic animals. Antibiotics.

[31] El-Hossary D., Mahdy A., Elariny E.Y., Askora A., Merwad A.M., Saber T., Dahshan H., Hakami N.Y., Ibrahim R.A. (2023). Antibiotic resistance, virulence gene detection, and biofilm formation in *Aeromonas* spp. isolated from fish and humans in Egypt. Biology.

[32] Huddleston J.R., Zak J.C., Jeter R.M. (2006). Antimicrobial Susceptibilities of *Aeromonas* spp. Isolated from Environmental Sources. Appl. Environ. Microbiol..

[33] Igbinosa E.O., Ogofure A.G., Beshiru A. (2022). Evaluation of different agar media for the antibiotic susceptibility testing of some selected bacterial pathogens. Univ. Lagos J. Basic Med. Sci..

[34] Miller R.A., Chuanchuen R., Gaunt P.S., Gandhi M., Burbick C.R., Gieseker C.M., Avendaño-Herrera R., Marchand S., Buller N., Smith P.R. (2020). Methods for Antimicrobial Broth Dilution and Disk Diffusion Susceptibility Testing of Bacteria Isolated from Aquatic Animals.

[35] Liu W., Li M., Xue M., Zhou Y., Jiang N., Meng Y., Liu Y., Jiang J., Liao X., Fan Y. (2024). Identification of *Aeromonas veronii* as the Pathogen Associated with Massive Mortality in Bronze Gudgeon (*Coreius heterodon*). Animals.

[36] Su Y., Zheng Y., Han Z., Liang E., Sun J. (2023). Identification and characterization of *Aeromonas veronii* and Vibrio cholerae from naturally diseased red swamp crayfish (*Procambarus clarkia*). Aquac. Rep..

[37] Sakulworakan R., Chokmangmeepisarn P., Dinh-Hung N., Sivaramasamy E., Hirono I., Chuanchuen R., Kayansamruaj P., Rodkhum C. (2021). Insight into whole genome of *Aeromonas veronii* isolated from freshwater fish by resistome analysis reveal extensively antibiotic resistant traits. Front. Microbiol..

[38] de Oliveira C.H., Moreno L.Z., Cardoso P.H., Silva A.P.S., Gomes V.T., Barbosa M.R., Balian S.C., Moreno A.M. (2024). Characterization of *Aeromonas* isolates from ornamental fish: Species, virulence genes, and antimicrobial susceptibility. Microorganisms.

[39] Leanovich S., Maksimyuk Y., Dziahtsiaryk S., Sidarenka A. (2025). Diversity, virulence factors and antibiotic resistance profiles of *Aeromonas* species isolated from farmed fish in Belarus. Microbe.

[40] Lampou E., Psychari E., Louka K., Kotzamanidis C., Malousi A., Petropoulos I., Kolygas M.N., Doukas D., Bitchava K. (2025). First Outbreak of Aeromoniasis, Caused by *Aeromonas veronii*, in Farmed European Seabass (*Dicentrarchus labrax*) in the Ionian Sea, Greece. Pathogens.

[41] Stephens S., Mahadevan R., Allen D.G. (2025). Establishing a quantitative link between crystal violet absorbance and biomass in biofilms. MethodsX.

[42] George T., Sivam V., Vaiyapuri M., Anandan R., Sivaraman G.K., Joseph T.C. (2025). Standardizing biofilm quantification: Harmonizing crystal violet absorbance measurements through extinction coefficient ratio adjustment. Arch. Microbiol..

[43] Xiong F., Cao L., Xiong J., Wu Y.F., Huang W.S., Chang M.X. (2022). Time-resolved and multi-tissue RNAseq provides new insights on the immune responses of European eels following infection with *Aeromonas hydrophila*. Water Biol. Secur..

[44] Lawal O.U., Bryan N., Parreira V.R., Anderson R., Chen Y., Precious M., Goodridge L. (2024). Phylogenomics of novel clones of *Aeromonas veronii* recovered from a freshwater lake reveals unique biosynthetic gene clusters. Microbiol. Spectr..

[45] Fernández-Bravo A., Figueras M.J. (2020). An update on the genus Aeromonas: Taxonomy, epidemiology, and pathogenicity. Microorganisms.

[46] Smyrli M., Triga A., Dourala N., Varvarigos P., Pavlidis M., Quoc V.H., Katharios P. (2019). Comparative study on a novel pathogen of European seabass. Diversity of *Aeromonas veronii* in the Aegean Sea. Microorganisms.

[47] Abdelsalam M., Elgendy M.Y., Elfadadny M.R., Ali S.S., Sherif A.H., Abolghait S.K. (2023). A review of molecular diagnoses of bacterial fish diseases. Aquac. Int..

[48] Silva-Santana G., de Sales F.L.S., Brandão M.L.L. (2026). Laboratory strategies for the identification of Burkholderia species: From classical phenotyping to advanced genomic and proteomic approaches. Antonie Leeuwenhoek.

[49] Ramesh M., Sen A., Vachher M., Nigam A. (2021). Delineating bacteria using DNA barcoding. Mol. Genet. Microbiol. Virol..

[50] Selim S., Harun-Ur-Rashid M., Jahan I., Elamir M.Y.M. (2025). Antibiotic susceptibility patterns of Aeromonas: Insights from aquatic ecosystems to public health impacts. Egypt. J. Aquat. Res..

[51] Embeti S., Gutta M., Gundi V.A. (2023). Antibiotics usage in aquaculture—An overview. Int. J. Pharm. Biol..

[52] Abu-Elala N., Abdelsalam M., Marouf S., Setta A. (2015). Comparative analysis of virulence genes, antibiotic resistance and gyrB-based phylogeny of motile *Aeromonas* species isolates from Nile tilapia and domestic fowl. Lett. Appl. Microbiol..

[53] Pei C., Song H., Zhu L., Qiao D., Yan Y., Li L., Zhao X., Zhang J., Jiang X., Kong X. (2021). Identification of Aeromonas veronii isolated from largemouth bass Micropterus salmoides and histopathological analysis. Aquaculture.

[54] Zeng Q., Liao C., Terhune J., Wang L. (2019). Impacts of florfenicol on the microbiota landscape and resistome as revealed by metagenomic analysis. Microbiome.

[55] Assane I.M., Gozi K.S., Valladão G.M.R., Pilarski F. (2019). Combination of antimicrobials as an approach to reduce their application in aquaculture: Emphasis on the use of thiamphenicol/florfenicol against *Aeromonas hydrophila*. Aquaculture.

[56] Grilo M.L., Sousa-Santos C., Robalo J., Oliveira M. (2020). The potential of *Aeromonas* spp. from wildlife as antimicrobial resistance indicators in aquatic environments. Ecol. Indic..

[57] Albini E., Caponi B., Pieralisi S., Orsini S., Blasi F., Massaccesi L., Maresca C., Scoccia E., Fiorucci A., Michelacci V. (2025). *Aeromonas* spp. as possible bacterial indicator for monitoring antibiotic resistance in seafood. Front. Microbiol..

[58] Nascimento M., Rodrigues J., Matias R., Jordao L. (2024). *Aeromonas* spp. in freshwater bodies: Antimicrobial resistance and biofilm assembly. Antibiotics.

[59] Uruén C., Chopo-Escuin G., Tommassen J., Mainar-Jaime R.C., Arenas J. (2020). Biofilms as promoters of bacterial antibiotic resistance and tolerance. Antibiotics.

[60] Chenia H.Y., Duma S. (2017). Characterization of virulence, cell surface characteristics and biofilm-forming ability of *Aeromonas* spp. isolates from fish and sea water. J. Fish Dis..

[61] De Silva L.A.D.S., Heo G.J. (2023). Biofilm formation of pathogenic bacteria isolated from aquatic animals. Arch. Microbiol..

[62] Prediger K.D.C., Dallagassa C.B., Moriel B., Vizzotto B.S., Volanski W., Souza E.M., Pedrosa F.O., Weiss V., Alberton D., Guizelini D. (2020). Virulence characteristics and antimicrobial resistance of *Aeromonas veronii* biovar sobria 312M, a clinical isolate. Braz. J. Microbiol..

[63] Cai Z., Guo L., Li J., Li H., Tang Y., Chi X., Liu Z., Ma X. (2025). Functional characterization of the global regulator Hfq in *Aeromonas veronii* reveals an essential role in pathogenesis and secretion of effectors. Microb. Pathog..

