# Supplementary material for: First Report and Integrated Characterization of Aeromonas veronii Associated with the Protected Fish Diptychus maculatus in Xinjiang, China
Source: Animals (Basel). 2026 Sep 4;16(17):2787. doi: 10.3390/ani16172787 (PMC13565465; doi:10.3390/ani16172787)
Supplement: Supplementary file 1 [file animals-16-02787-s001.zip › animals-4430251-supplementary.pdf]

animals

Supplementary Materials

First Report and Integrated Characterization of *Aeromonas veronii* Associated with the Protected Fish *Diptychus maculatus* in Xinjiang, China

Supplementary Table S1. Metadata and traceability information of *Aeromonas veronii* isolates recovered from individual *Diptychus maculatus* specimens.

Fish ID	Isolate ID	Sampling date	Tissue source	Sex	Total length (mm)	Body weight (g)
DM-F01	DMNZ01	June 2024	Eye	Female	169.96	25.67
DM-F02	DMNZ02	June 2024	Intestine	Male	113.68	8.62
DM-F03	DMNZ03	June 2024	Dorsal fin	Male	114.77	8.27
DM-F04	DMNZ04	June 2024	Body kidney	Undetermined	98.55	5.71
DM-F05	DMNZ05	June 2024	Skin	Female	100.04	5.85
DM-F06	DMNZ06	June 2024	Gill	Not recorded	155.05	24.21
DM-F07	DMNZ07	June 2024	Gonad	Male	116.89	9.50
DM-F08	DMNZ08	June 2024	Spleen	Male	101.37	6.02

Supplementary Table S2. Raw inhibition zone diameters (mm) of *Aeromonas veronii* isolates (DMNZ01–DMNZ08) and reference strain obtained from triplicate antimicrobial susceptibility assays.

Antibiotic	Disc content	DMNZ01	DMNZ02	DMNZ03	DMNZ04	DMNZ05	DMNZ06	DMNZ07	DMNZ08	BNCC13846
**Enrofloxacin**	**5 μg**	16.9 ± 0.20	17.4 ± 0.10	17.0 ± 0.10	17.3 ± 0.15	17.5 ± 0.10	17.4 ± 0.15	17.2 ± 0.11	17.0 ± 0.11	19.0 ± 0.11
**Ciprofloxacin**	**5 μg**	13.6 ± 0.15	14.5 ± 0.11	13.7 ± 0.15	13.6 ± 0.17	14.5 ± 0.10	14.8 ± 0.17	14.5 ± 0.10	14.0 ± 0.11	24.36 ± 0.11
**Norfloxacin**	**10 μg**	12.3 ± 0.10	12.4 ± 0.17	12.0 ± 0.10	12.3 ± 0.15	12.5 ± 0.10	12.2 ± 0.17	12.3 ± 0.11	12.4 ± 0.11	18.8 ± 0.11
**Ofloxacin**	**5 μg**	13.6 ± 0.17	13.5 ± 0.20	13.7 ± 0.20	14.0 ± 0.20	13.4 ± 0.21	14.5 ± 0.20	13.5 ± 0.18	14.6 ± 0.17	16.24 ± 0.11
**Neomycin**	**30 μg**	11.0 ± 0.14	11.1 ± 0.20	11.3 ± 0.20	11.0 ± 0.20	11.2 ± 0.20	10.9 ± 0.15	11.9 ± 0.18	11.0 ± 0.20	14.68 ± 0.15
**Penicillin**	**10 IU**	5.8 ± 0.15	6.0 ± 0.20	5.7 ± 0.12	5.8 ± 0.15	5.9 ± 0.13	5.5 ± 0.12	5.7 ± 0.20	5.8 ± 0.15	7.0± 0.11
**Amoxicillin**	**10 μg**	5.8 ± 0.10	6.3 ± 0.11	5.9 ± 0.15	6.1 ± 0.13	6.0 ± 0.20	5.5 ± 0.17	6.1 ± 0.17	6.2 ± 0.10	8.0± 0.11
**Cefotaxime**	**30 μg**	28.0 ± 0.20	27.8 ± 0.15	27.9 ± 0.20	28.0 ± 0.20	27.9 ± 0.18	28.7 ± 0.17	28.0 ± 0.20	27.5 ± 0.20	29.4± 0.17
**Ceftriaxone**	**30 μg**	24.0 ± 0.20	24.1 ± 0.20	23.9 ± 0.10	23.8 ± 0.15	24.1 ± 0.17	24.2 ± 0.20	23.9 ± 0.18	23.7 ± 0.21	29.6± 0.15
**Tetracycline**	**30 μg**	11.4 ± 0.14	11.5 ± 0.15	10.8 ± 0.15	11.4 ± 0.17	11.4 ± 0.15	11.6 ± 0.16	10.9 ± 0.15	11.5 ± 0.14	16.53± 0.12
**Doxycycline**	**30 μg**	17.3 ± 0.15	16.9 ± 0.15	17.4 ± 0.14	17.1 ± 0.14	17.0 ± 0.15	17.5 ± 0.17	17.3 ± 0.17	17.4 ± 0.15	23.67 ± 0.10
**Oxytetracycline**	**30 μg**	16.7 ± 0.20	16.8 ± 0.17	16.3 ± 0.20	16.0 ± 0.20	15.9 ± 0.18	16.9 ± 0.20	16.0 ± 0.18	15.8 ± 0.20	20.69± 0.15
**TMP-SMX**	**1.25/23.75 μg**	14.6 ± 0.10	14.5 ± 0.12	14.7 ± 0.10	14.6 ± 0.11	14.8 ± 0.13	14.5 ± 0.13	14.6 ± 0.12	14.7 ± 0.10	20.4± 0.10
**Erythromycin**	**15 μg**	10.9 ± 0.11	11.3 ± 0.10	11.0 ± 0.11	10.7 ± 0.15	11.4 ± 0.10	10.5 ± 0.11	11.0 ± 0.11	11.3 ± 0.13	19.0± 0.14
**Florfenicol**	**30 μg**	21.0 ± 0.12	20.9 ± 0.14	21.3 ± 0.14	20.8 ± 0.15	21.0 ± 0.12	20.1 ± 0.15	21.0 ± 0.15	21.7 ± 0.14	30.7± 0.20
